# Bidirectional Effect of IFN-γ on Th17 Responses in Experimental Autoimmune Uveitis

**DOI:** 10.3389/fopht.2022.831084

**Published:** 2022-03-07

**Authors:** Hui Shao, Henry J. Kaplan, Deming Sun

**Affiliations:** ^1^ Department of Ophthalmology and Visual Sciences, Kentucky Lions Eye Center, University of Louisville, Louisville, KY, United States; ^2^ Department of Ophthalmology, Saint Louis University School of Medicine, Saint Louis, MO, United States; ^3^ Doheny Eye Institute and Department of Ophthalmology, David Geffen School of Medicine at University of California, Los Angeles, Los Angeles, CA, United States

**Keywords:** IFN-gamma, experimental autoimmune uveitis, gamma delta T cell, Th17, autoimmunity

## Abstract

Pro- and ant-inflammatory effects of IFN-γ have been repeatedly found in various immune responses, including cancer and autoimmune diseases. In a previous study we showed that the timing of treatment determines the effect of adenosine-based immunotherapy. In this study we examined the role of IFN-γ in pathogenic Th17 responses in experimental autoimmune uveitis (EAU). We observed that IFN-γ has a bidirectional effect on Th17 responses, when tested both *in vitro* and *in vivo*. Anti-IFN-γ antibody inhibits Th17 responses when applied in the initial phase of the immune response; however, it enhances the Th17 response if administered in a later phase of EAU. In the current study we showed that IFN-γ is an important immunomodulatory molecule in γδ T cell activation, as well as in Th17 responses. These results should advance our understanding of the regulation of Th17 responses in autoimmunity.

## Highlights

* IFN-γ has a bidirectional effect on Th17 responses
* Anti-IFN-γ antibody inhibits early inflammatory responses but enhances later responses
* Effect of IFN-γ on γδ T cells is closely associated with an altered Th17 response

## Introduction

IFN-γ production is a hallmark of the T helper (Th)1 response (1, 2). As a key player in cellular immunity, IFN-γ is capable of orchestrating numerous immune responses in infections and cancers. It immunomodulates antigen processing and presentation (3), increases leukocyte trafficking (4), induces anti-viral responses (5), boosts anti-microbial functions (6, 7) and affects cellular proliferation and apoptosis (8). Although the major source of IFN-γ in adaptive immune responses is T cells (9), various innate cells such as NK/NKT cells (2, 10), macrophages (11, 12), dendritic cells (13–15) and γδ T cells (16, 17) are capable of producing IFN-γ. Indeed, in the initiation stage of autoimmune diseases, innate cells such as NK cells are the main source of IFN-γ production (18), albeit transiently (2).

IFN-γ has either pro- or anti-inflammatory effects in various immune responses, including cancer and autoimmunity (18–22). The earlier finding that IFN-γ inhibits Th17 responses (23, 24) suggested that neutralization of IFN-γ would elicit stronger Th17 responses (25, 26). Because we previously found that adenosine-based immunotherapies are strictly “timing dependent” (27, 28), we wished to investigate whether immunomodulation by treatments other than adenosine or adenosine receptors also might depend on the timing of treatment. In our previous study, we found that treatment of mice with induced experimental autoimmune uveitis (EAU) with adenosine deaminase (ADA), an enzyme that degrades adenosine, inhibited Th17 pathogenic T cell responses and suppressed EAU (27). The inhibitory effect of ADA was restricted to the active stage of disease; but ADA was ineffective if administered during the quiescent disease stage (27). Likewise, treatment of EAU-induced mice with an antagonist specific for adenosine receptor A2AR only inhibited EAU if given during the active phase of intraocular inflammation (28). We investigated the effect of adding anti-IFN-γ antibody to *in vitro* responding T cells and EAU-induced mice at different time points to determine whether IFN-γ has a regulatory effect on Th17 responses, and whether a similar timing effect might be identified. Our results demonstrated that anti-IFN-γ antibody inhibited Th17 responses if provided during the initial phase of T cell activation (i.e., the early phase of EAU induction). However, the effect was reversed once T cell activation was initiated *in vitro* or when anti-IFN-γ antibody was administered to EAU-induced mice in a later phase of EAU induction.

Our previous studies showed that activation of γδ T cells closely correlated with augmented Th17 responses (29–32). Therefore, we also examined the effect of anti-IFN-γ treatment on γδ T cell activation. Anti-IFN-γ treatment during the early phase of EAU induction inhibited γδ T cell activation, whereas treatment during a later phase of EAU induction enhanced γδ T cell activation similar to the Th17 responses. Thus, in the current study we showed a “timing effect” for IFN-γ similar to that of adenosine-based immunotherapy in EAU (27, 33).

## Materials and Methods

### Animals and Reagents

Female C57BL/6 (B6), TCR-δ^-/-^, and IFN-γ^-/-^ mice were purchased from Jackson Laboratory (Bar Harbor, ME, USA); 12- to 16-week-old mice were used in all studies. All mice were housed and maintained in the animal facilities of the University of California Los Angeles. All protocols in this study were approved by the Committee on the Ethics of Animal Experiments of University of California, Los Angeles (IACUC permit number: ARC#2014-029-03A), in compliance with the Guide for the Care and Use of Laboratory Animals published by the US National Institutes of Health.

Recombinant murine IFN-γ, and IL-23 were purchased from R & D Systems (Minneapolis, MN, USA). FITC-, PE-, or allophycocyanin-conjugated Abs against mouse CD3, CD4, Foxp3, αβ TCR, or γδ TCR (GL3) and their isotype control Abs were purchased from Biolegend (San Diego, CA, USA). PE-conjugated anti-mouse IFN-γ (XMG1.2), IL-17 (TC11-18H10.1) Abs were purchased from Santa Cruz Biotechnology (Dallas, TX, USA).

### EAU Induction and Anti-IFN-γ Treatment

EAU was induced in B6 mice by s.c. injection of 200 μl of emulsion containing 200 μg of human interphotoreceptor retinoid-binding protein (IRBP)_1-20_ (Sigma-Aldrich, St. Louis, MO, USA) in CFA (Difco, Detroit, MI, USA) at six spots at the tail base and on the flank, and by i.p. injection with 300 ng of pertussis toxin. Mice were then examined three times a week until the end of the experiment (d 30 post-immunization).

**Scheme 1:**
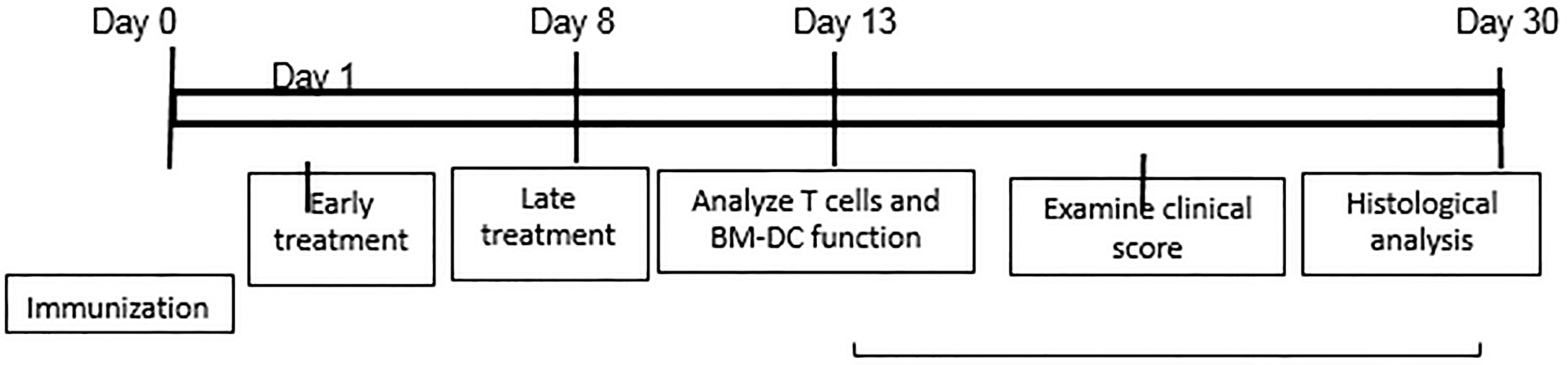
Schematic experimental procedure.

For *in vivo* administration of IFN-γ, immunized B6 mice were randomly divided into three groups (n=6), one of which received an i.p. injection of anti-IFN-γ (100 μg/mouse) at d 0 (day of immunization and the second at d 8 post-immunization. The mice in the control group received PBS. At d 13 post-immunization (the time at which the highest T cell response is seen), responder T cells were purified from the spleen and draining lymph nodes stimulated *in vitro* with the immunizing peptide and APCs (irradiated spleen cells) under culture conditions that favor Th17 or Th1 autoreactive T cell expansion (medium containing 10 ng/ml of, respectively, IL-23 or IL-12) (24, 25) A schematic procedure of disease induction and examination of mice under investigation is shown in **Scheme 1**.

### Adoptive Transfer Assay Testing Uveitogenic Activity of IRBP-Specific T Cells

IRBP-specific T cells were prepared as we previously described (29). Briefly, CD3^+^ T cells from IRBP_1-20_/CFA-immunized B6 mice were isolated 13 d postimmunization. Then 1 x 10^7^ cells in 2 ml of RPMI medium in a 6-well plate (Costar) were stimulated with 20 μg/ml of IRBP_1-20_ in the presence of 1 x 10^7^ irradiated syngeneic spleen cells as APCs. After 2 d, the activated lymphoblasts were isolated by gradient centrifugation on Lymphoprep (Robbins Scientific, Mountain View, CA, USA). The pathogenic activity was determined after transfer of the IRBP-specific T cells to the naïve B6 recipient mice *via* i.p. injection (2 x 10^6^/mouse).

### EAU Evaluation

The mice were examined three times a week for 30 d post-immunization. The clinical signs of EAU were evaluated using fundoscopic examination. Fundoscopic grading of disease was performed using the scoring system described previously (34).

At 30 d post-immunization, the mice were euthanized, and the eyes were collected for histological examination. For histology, whole eyes were collected at the end of the experiment and prepared for histopathological evaluation. The eyes were immersed for 1 h in 4% phosphate-buffered glutaraldehyde, then transferred to 10% phosphate-buffered formaldehyde until they were processed. Fixed and dehydrated tissues were embedded in methacrylate, and 5 μM sections were cut through the pupillary-optic nerve plane and stained with H&E. The eyes were fixed overnight at 40C in Davison’s solution and then processed as paraffin-embedded blocks.

### T Cell Preparation

Responder CD3^+^ T cells were purified from B6 mice immunized with the human IRBP_1-20_ peptide (29, 31, 35). Nylon wool-enriched splenic T cells from naive or immunized mice were incubated sequentially for 10 min at 4°C with FITC-conjugated anti-mouse γδ TCR or αβ TCR Abs and for 15 min at 4°C with anti-FITC Microbeads (Miltenyi Biotec GmbH, Bergisch Gladbach, Germany). The cells were then separated into bound and non-bound fractions on an autoMACS™ separator column (Miltenyi Biotec GmbH). To obtain a sufficient number of cells, we routinely pool the cells obtained from all six mice in the same group, before the T cells are further enriched using MACS column. The purity of the isolated cells, as determined by flow cytometric analysis using PE-conjugated Abs against αβ or γδ T cells, was >95%.

### Assessment of Th1 and Th17 Polarized Responses

Responder CD3^+^ T cells (3 x 10^6^) were co-cultured for 48 h with IRBP_1-20_ (10 µg/ml) and with irradiated spleen cells (2 x 10^6^/well) as APCs in a 12-well plate under either Th17 polarized conditions (culture medium supplemented with 10 ng/ml of IL-23) or Th1 polarized conditions (culture medium supplemented with 10 ng/ml of IL-12). The responder αβ T cells were collected from IRBP_1-20_-immunized B6 mice, on d 13 post-immunization. Forty-eight hours after stimulation, IL-17 and IFN-γ levels in the culture medium were measured using ELISA kits (R & D Systems) and the percentage of IFN-γ^+^ and IL-17^+^ T cells among the responder T cells was determined by intracellular staining after 5 d of culture, followed by FACS analysis, as described below (31, 36).

### Immunofluorescence Flow Cytometry for Surface and Cytoplasmic Antigens

*In vivo* primed T cells were stimulated with the immunizing antigen and APCs for 5 d. The T cells were then separated using Ficoll gradient centrifugation and stimulated *in vitro* for 4 h with 50 ng/ml of PMA, 1 μg/ml of ionomycin, and 1 μg/ml of brefeldin A (all from Sigma). Aliquots of cells (2 x 105 cells) were incubated for 30 min at 4°C with anti-αβ or -γδ TCR antibodies, then fixed, permeabilized overnight with Cytofix/Cytoperm buffer (eBioscience, San Diego, CA, USA), and intracellularly stained with PE-conjugated anti-mouse IFN-γ antibodies or FITC-labeled anti-mouse IL-17 antibodies. Data collection and analysis were performed on a FACScalibur flow cytometer using CellQuest software.

### Cytokine Assays by ELISA

Cytokine (IL-17 and IFN-γ) levels in the culture medium were measured using ELISA kits (R & D) following manufacturer’s instructions.

### Statistical Analysis

The results in the figures are representative of one experiment, which was repeated 3-5 times. The statistical significance of differences between groups in a single experiment was initially analyzed by 2-way Student’s t-tests, and if statistical significance was detected, the Student–Newman–Keuls *post-hoc* test was subsequently used. A P value of less than 0.05 was considered a statistically significant difference and marked with one *; when P<0.01, two ** were used.

## Results

### Anti-IFN-γ Affects Th1 and Th17 Responses Differently *In Vitro*

**Figure 1:**
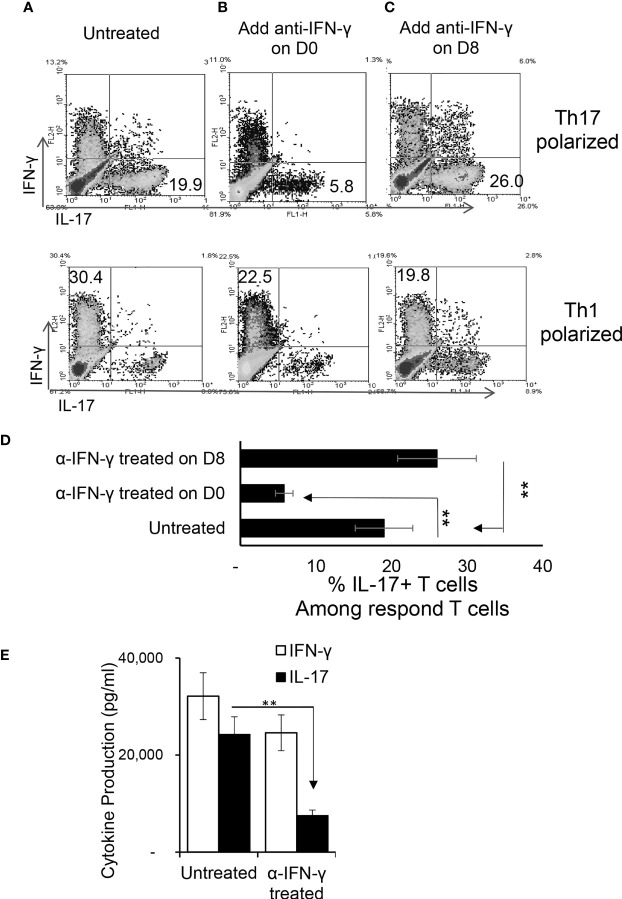
Effect of anti-IFN-γ on *in vitro* Th1 and Th17 responses. Purified CD3^+^ T cells were stimulated with the immunizing antigen and irradiated splenic APCs, under Th1- (culture medium containing IL-12) or Th17 (culture medium containing IL-23) polarized condition and in the absence or presence of anti-IFN-γ antibody (5 μg/ml). The abundance of IFN-γ^+^ and IL-17^+^ T cells among the responder T cells was estimated after intracellular staining with fluorescence labeled anti-IFN-γ or anti–IL-17 antibodies, 5 d after *in vitro* stimulation. Compare to untreated **(A)**, anti-IFN-γ inhibits T cell activation when added on d 0 **(B)** but enhances T cell activation if added on d 8 **(C)**. The results in **(A–C)** are from a single experiment and pooled results of three separate studies are shown in **(D)**. **p < 0.01. The IL-17 and IFN-γ levels in the culture medium were measured by ELISA after anti-IFN-γ Ab added on d0 **(E)**. The data are pooled from three independent experiments. **p < 0.01.

To determine the effect of IFN-γ on autoreactive antigen specific T cell responses *in vitro*, CD3^+^ T cells from the spleen and draining lymph nodes of B6 mice were isolated at the height of induced responses, 13 d after immunization with IRBP_1-20._ They were stimulated with the immunizing antigen and irradiated splenic APCs under Th1- or Th17- polarized conditions, with/without anti-IFN-γ (d 0). As demonstrated in **Figures 1A, B**, anti-IFN-γ treated on day 0 (day of immunization) inhibited the response of Th17 cells to a much greater extent than it inhibited the response of Th1 cells; similarly, IL-17 production was decreased much more than production of IFN-γ (**Figure 1E**). The effect of anti-IFN-γ was reversed when the antibody was added 8 d post-stimulation (**Figure 1C**): with the Th17 cell and IL-17 responses enhanced. The effect on Th1 cells or IFN-γ production was minimal.

### Anti-IFN-γ Affects Th1 and Th17 Responses Differently *In Vivo*

**Figure 2:**
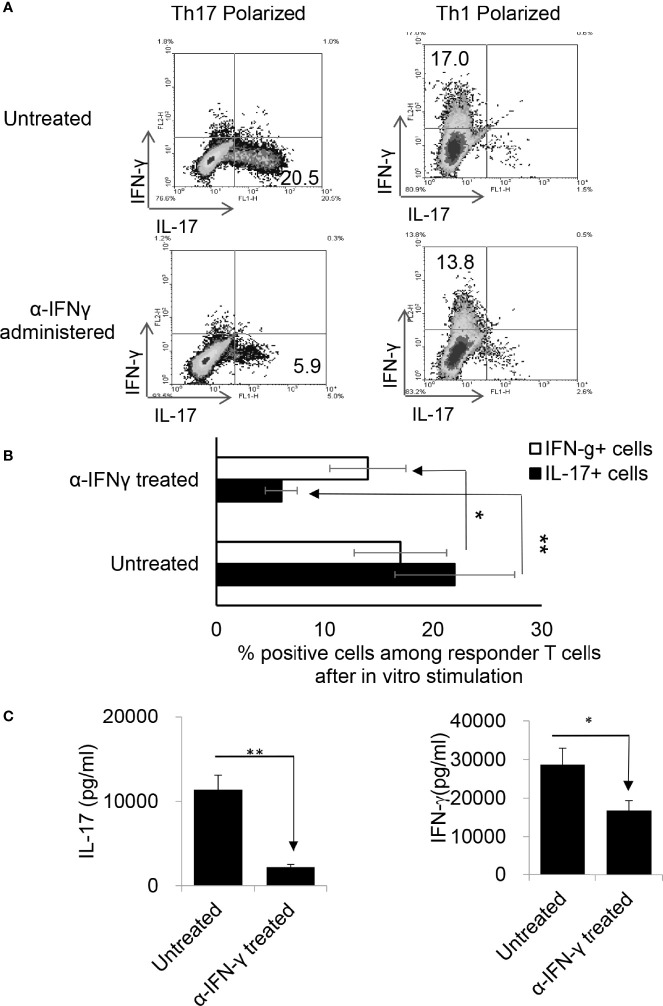
Anti-IFN-γ inhibited Th17 responses *in vivo.* Anti-IFN-γ on d0 (the day of immunization) preferentially inhibited Th17 cells. Two groups of B6 mice (n = 6) were immunized with IRBP_1-20_/CFA; one was injected with anti-IFN-γ (100cμg/mouse) *via* i.p. and the group received PBS. Thirteen days post-immunization, mice were sacrificed and the number of Th1 and Th17 cells among responder T cells were assessed 5 d after *in vitro* stimulation after intracellular staining with anti-IL-17 and anti-IFN-γ antibody. The results in **(A)** are from a single experiment and pooled results of three separate studies are shown in **(B)**. Responder T cells of B6 mice administered with anti-IFN-γ produced significantly decreased levels of IL-17. The IL-17 and IFN-γ levels in the culture supernatants were measured by ELISA after stimulation of responder T cells with the immunizing antigen and splenic APCs for 2 (d) The data are pooled from three independent experiments. *P < 0.05 and **P < 0.01.

To determine whether anti-IFN-γ antibody would have a similar effect *in vivo*, two groups of B6 mice (n=6) were immunized with IRBP_1-20_ with or without an i.p. injection of anti-IFN-γ (100μg/mouse) on the day of immunization (d 0). Thirteen days post-immunization the mice were sacrificed and Th1 and Th17 responses were assessed. **Figure 2** shows that anti-IFN-γ antibody again significantly inhibited the Th17 response while inhibiting the Th1 response only slightly, similar to its effects seen *in vitro*.

### The Effect of Anti-IFN-γ on EAU Development and the Th17 Response Depends on Timing

**Figure 3:**
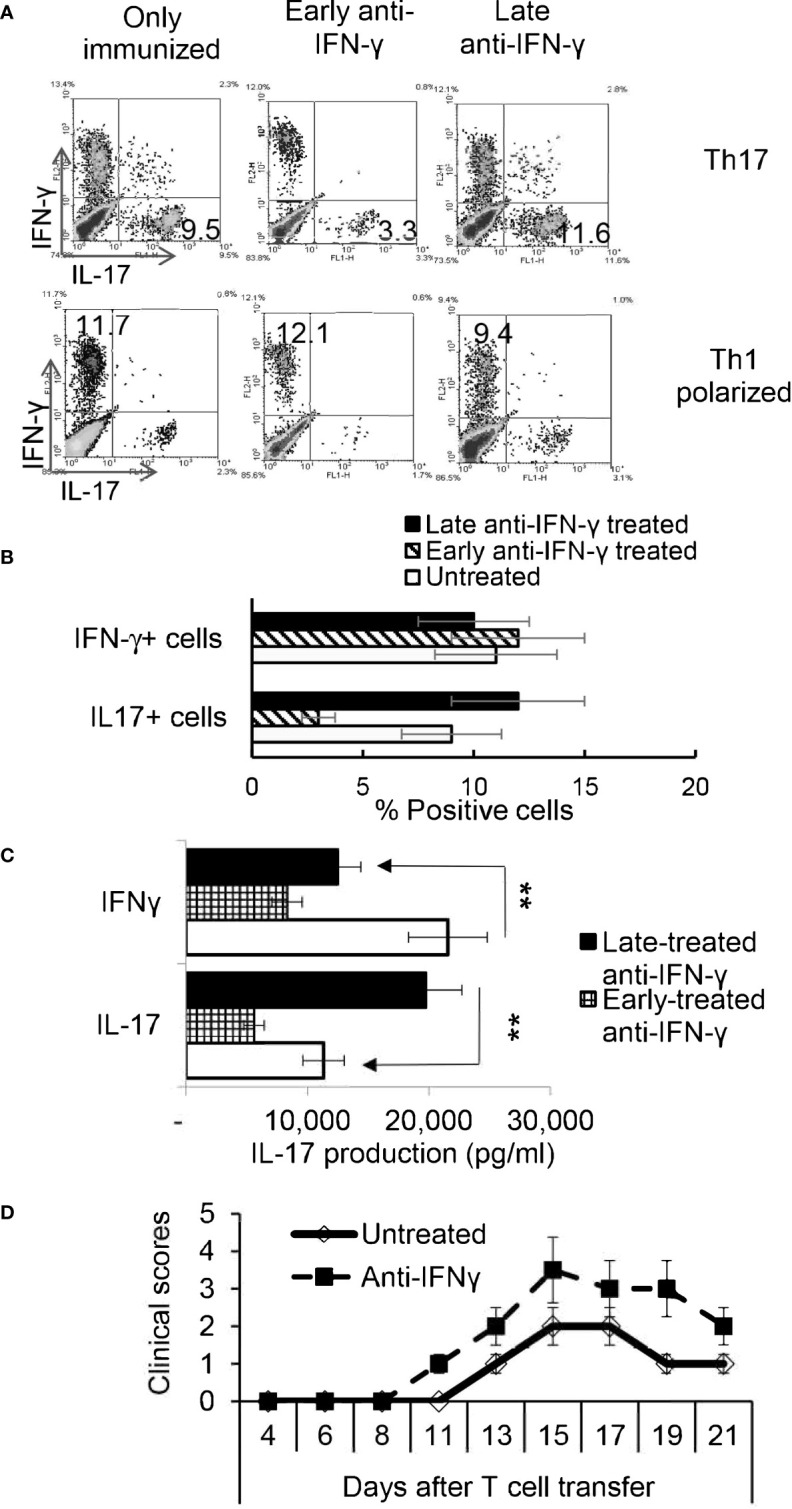
Effect of anti-IFN-γ administration is “timing dependent”. Th17 responses were significantly decreased in mice early treated with anti-IFN-γ but were enhanced in mice late treated with anti-IFN-γ. Groups of B6 mice (n=6) immunized with IRBP1-20/CFA received anti-IFN-γ on d0 or d8. Thirteen days post immunization CD3^+^ responder T cells were stimulated *in vitro* with the immunizing peptide and APCs, under Th1- or Th17-polarized conditions. 5 d after *in vitro* stimulation the number of IFN-γ^+^ and IL-17^+^ T cells was estimated after intracellular staining. The results from **(A)** are from a single experiment and pooled results of three separate studies are shown in **(B)**. **P < 0.01. B) Cytokine production by Th17 cells was enhanced by late anti-IFN-γ treatment. The IL-17 and IFN-γ levels in the culture medium were measured by ELISA after stimulation of responder T cells with the immunizing antigen and splenic APCs for 2 d **(C)**. **P < 0.01. IRBP-specific T cells were separated from IRBP-immunized mice with or without an anti-IFN-γ administration on day 13 of immunization. After 2 d *in vitro* stimulation with the immunizing antigen and splenic APCs, under Th17-polarizing conditions, the activated T cells were adoptively transferred to naive B6 mice (2 x 10^6^/mouse) *via* i.p. injection and clinical expression of EAU was scored **(D)**.

To determine whether the timing effect observed *in vitro* also applied *in vivo*, groups of B6 mice (n=6) were immunized with IRBP_1-20_/CFA. Because preliminary testing revealed that results differed when anti-IFN-γ was injected between d 0 to 3 vs d 6 to 8 post immunization, two time points were selected for subsequent studies. Those mice injected on the day of immunization (d0) were designated as “early-treated”, and those injected on day 8 after immunization were designated as “late-treated”. As shown in **Figures 3A, B**, the number of IL-17^+^ cells among the responder T cells in early-treated mice was significantly decreased (from 9.5% in the control group to 3.3% in the treated group), whereas this number was significantly increased in late-treated mice (from 9.5% in the control group to 11.6% in the treated group) (**Figure 3A**, upper panels). In contrast, numbers of IFN-γ^+^ T cells changed little between early- and late-treated mice (**Figure 3A**, lower panels). Cytokine production (**Figure 3C**) confirmed intracellular staining in that mice in the late group produced more IL-17, whereas those in the early group produced less IL-17. *In vivo* administration of anti-IFN-γ had a stronger inhibitory effect on IFN-γ production of responder T cells, when compared with *in vitro* tests (**Figure 1**), regardless of whether the antibody was injected early or late. The difference between these results and the intracellular staining suggests that Th1 responses are modestly inhibited in treated mice.

### IL-17^+^ T Cells Isolated From Late IFN-γ Treated Recipients Are More Pathogenic

To determine whether pathogenic IRBP-specific IL-17^+^ T cell responses were enhanced in mice that received late treatment with anti-IFN-γ we performed adoptive transfer tests. IL-17^+^ T cells from IRBP-immunized mice administered anti-IFN-γ on d 8 were compared to PBS injected mice (i.e. the control) on d8. 2 x 10^6^ IRBP-specific IL-17^+^ cells were adoptively transferred to naive B6 mice by i.p. injection after 2 d of *in vitro* stimulation with the immunizing antigen and splenic APCs, under Th17-polarizing conditions. Adoptively transferred Th17 cells from late anti-IFN-γ treated mice induced significantly more severe EAU in recipient mice when compared to controls (**Figure 3D**). The total number of IL-17^+^ T cells was also significantly greater in anti-IFN-γ treated mice (data not shown).

### Similar Timing Effect Exists in γδ T Cell Responses in Anti-IFN-γ Treated Mice

**Figure 4:**
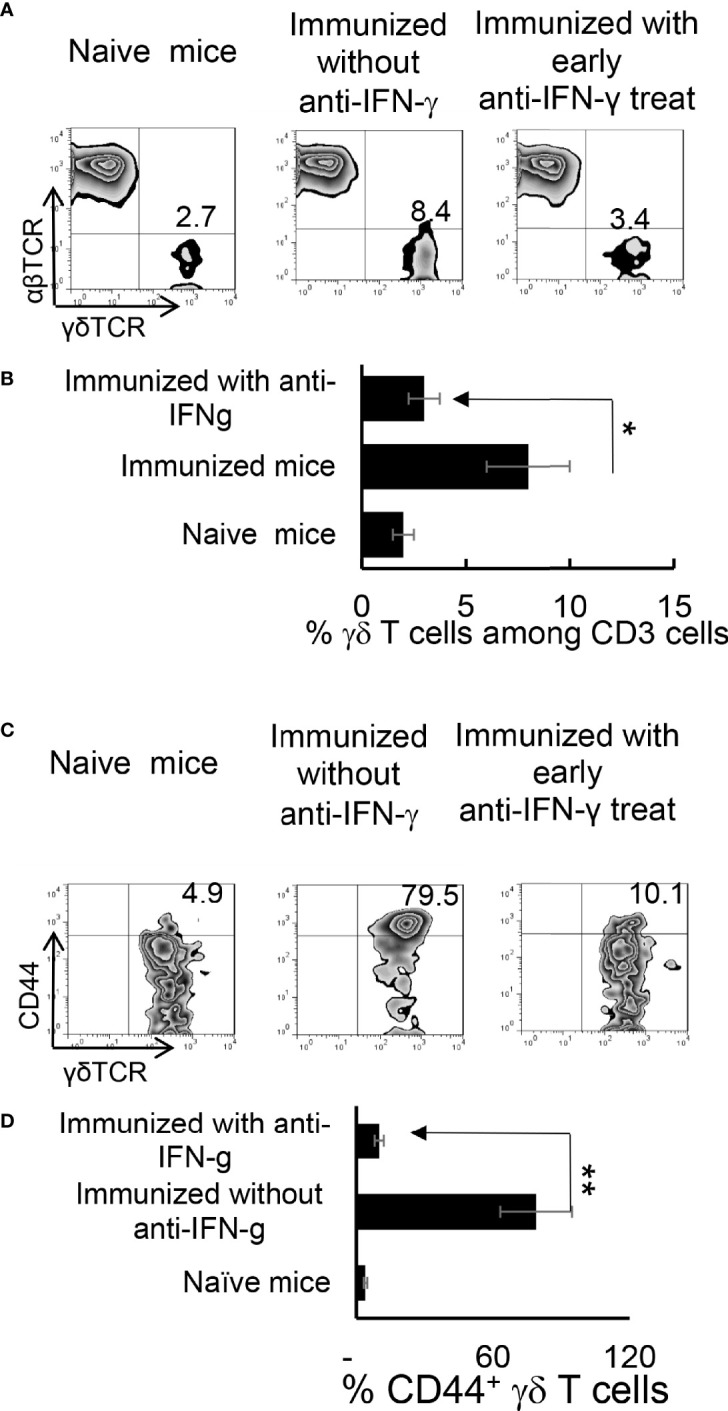
Altered γδ T cell responses in anti-IFN-γ treated mice. γδ T cell activation and expansion was inhibited in early anti-IFN-γ treated mice. Freshly isolated CD3^+^ cells from naïve and immunized B6 mice, with or without anti-IFN-γ administration (day 0), were stained with PE-anti-αβTCR and FITC-anti-γδTCR antibodies before they were subjected to FACS analysis **(A)**. They were also dually stained with PE-anti-mouse CD44 and FITC-anti-γδTCR antibodies **(C)**. A summary of multiple assays is shown in **(B, D)**. *P < 0.05, **P < 0.01.

**Figure 5:**
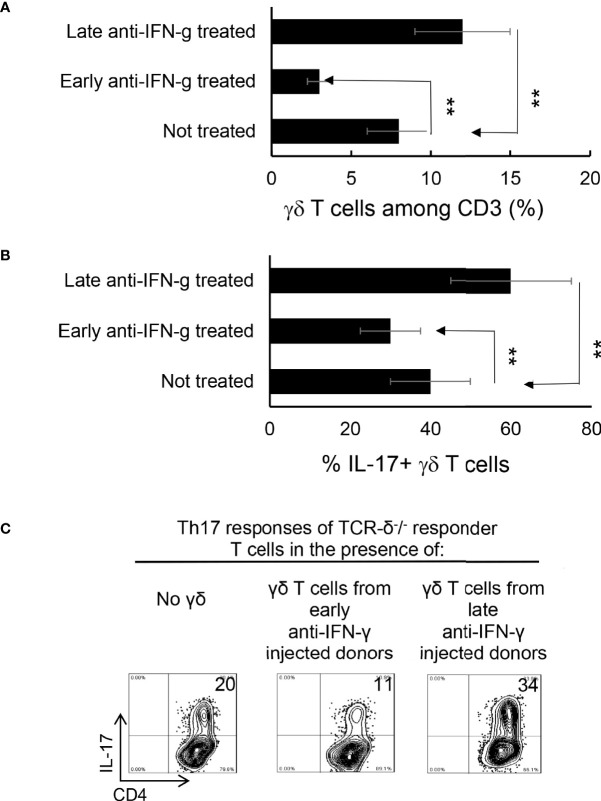
γδ T cells isolated from late anti-IFN-γ treated mice have enhanced pro-Th17 activity.CD3^+^ responder T cells (1 5 x 10^6^/well) isolated from immunized TCR-δ^-/-^ mice, with or without a prior anti-IFN-γ administration were stimulated with the immunizing antigen and irradiated splenic APCs, under Th17 polarized condition. Total γδ T cell numbers **(A)**, as well as proportional number of IL-17^+^ versus total γδ T cells **(B)** were compared. **P < 0.01. *In vitro* Th17 response of TCR-δ^-/-^ responder T cells were assessed with or without an addition of γδ T cells isolated from early or late treated anti-IFN-γ mice **(C)**. The number of IL-17^+^ T cells among the gated CD4^+^ responder T cells was estimated after intracellular staining with anti–IL-17 antibody, 5 d after *in vitro* stimulation.

We have previously shown that γδ T cells regulate Th17 responses (29, 31), whereby activation of γδ T cells augmented Th17 reactivity (30–32). Therefore, we thought it important to examine the state of γδ activation after anti-IFN-γ treatment. In early anti-IFN-γ treated mice, the frequency of γδ T cells among total CD3^+^ T cells was significantly reduced (**Figures 4A, B**), suggesting that IFN-γ is required for early-stage γδ cell proliferation. Perhaps more importantly, the ratio between CD44^+^ and CD44^-^ γδ T cells was greatly decreased (**Figures 4C, D**), indicating that neutralizing IFN-γ at early stages disease had an inhibitory effect on γδ activation *in vivo*. By contrast, the number of γδ T cells were significantly increased among total CD3^+^ T cells in mice that received late anti-IFN-γ treatment (**Figure 5A**) compared to early anti-IFN-γ treated or untreated B6 mice. Moreover, the number of IL-17^+^ among total γδ T cells was significantly higher in mice that received late anti-IFN-γ treatment (**Figure 5B**).

### γδ T Cells Separated From Late Anti-IFN-γ Treated Mice Enhance Th17 Activity *In Vitro*

To further investigate if the enhanced γδ activation in mice with late anti-IFN-γ treatment contributed to augmented Th17 responses, we compared the *in vitro* Th17 response of TCR-δ^-/-^ responder T cells with or without the addition of γδ T cells from early or late treated anti-IFN-γ mice (30–32). **Figure 5C** shows that the Th17 response of the TCR-δ^-/-^ responder T cells were significantly higher after adding γδ T cells (5% of total CD3^+^ cells) isolated from late anti-IFN-γ treated mice (right panel) compared to those without added γδ T cells (left panel) or with added γδ T cells isolated from early anti-IFN-γ treated mice (middle panel).

### Augmented Th17 Responses in IFN-γ^-/-^ Mice Are Contributed by Multiple Aberrant Immune Responses

**Figure 6:**
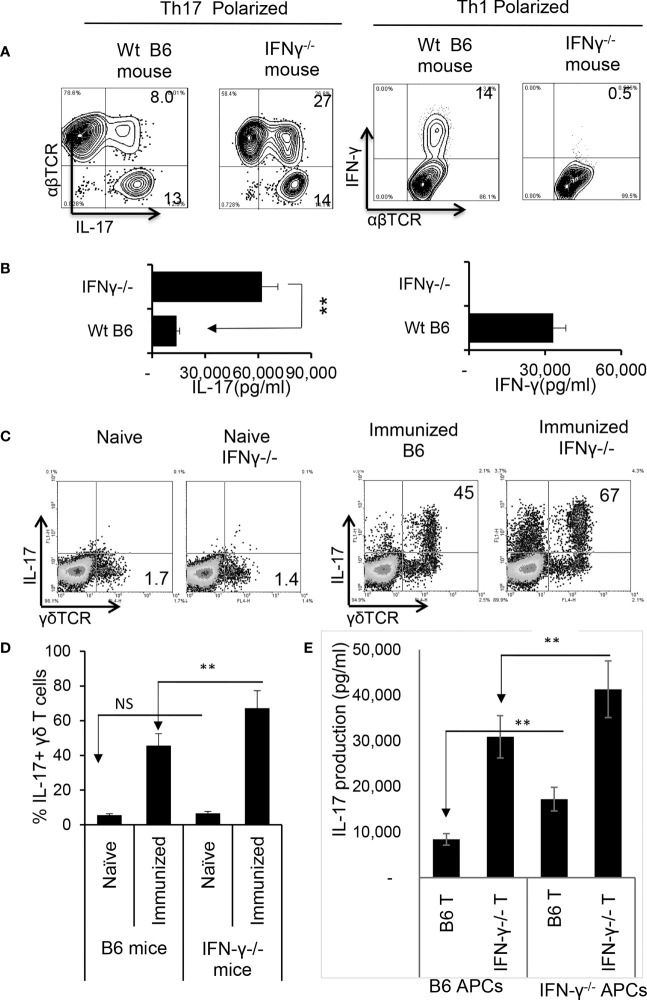
Augmented Th17 responses in IRBP_1-20_/CFA-immunized IFN-γ^-/-^ mice. CD3^+^ responder T cells were purified from IRBP_1-20_/CFA-immunized B6 and IFN-γ^-/-^ mice. They were stimulated *in vitro* with the immunizing peptide and APCs, under Th17 or Th1 polarized conditions as indicated. After 5 d of *in vitro* stimulation, Th1 and Th17 responses specific for the immunizing antigen were estimated by assessing IFN-γ^+^ and IL-17^+^ T cells intracellularly stained with fluorescence -labeled anti-IFN-γ or anti–IL-17 antibodies **(A)**. Immunized IFN-γ-/- mice also produced increased IL-17 compared to B6 mice. Production of IFN-γ and IL-17 by the B6 or IFN-γ-/- responder T cells after 48 h antigen stimulation *in vitro* was assessed by ELISA. Data are pooled from three independent experiments are shown **(B)**. **P < 0.01. Number of γδ T cells and IL-17 secretion from immunized IFN-γ^-/-^ mice exceeded that of immunized wt B6 mice. CD3^+^ T cells isolated from naïve, immunized B6 and immunized IFN-γ^-/-^ mice were stained with anti-IL-17 and anti-γδTCR, before FACS analysis **(C)**. The data from three independent experiments is shown in **(D)**. **P < 0.01. NS, not significant. Contribution of antigen-specific T cells and the effect of splenic APCs in augmented Th17 responses were examined. IFN-γ^-/-^ and B6 mice responder T cells (1.5 x 10^6^/well) from IFN-γ^-/-^ or B6 mice were stimulated by B6 splenic APCs (**E**, left panels) or IFN-γ^-/-^ splenic APCs (**E**, right panels). IL-17 in culture supernatants were assessed by ELISA harvested 48 h after *in vitro* stimulation. The data are pooled from three independent experiments. **P < 0.01.

Finally, we compared the immune response between IFN-γ-deficient and wt-B6 mice, as a further means to better understand the role of IFN-γ in pathogenic Th17 responses. **Figure 6A** shows that a greater number of responder T cells expressed IL-17 in IFN-γ^-/-^ mice compared to B6 mice (**Figure 6A**) when CD3^+^ T cells were stimulated with the immunizing antigen and APCs for 5 d; additionally, IFN-γ^-/-^ T cells produced significantly higher amounts of IL-17 (**Figure 6B**). The number of γδ T cells among the CD3^+^ splenic cells did not differ significantly between unimmunized IFN-γ^-/-^ and B6 mice; however, the total number of γδ T cells, as well as numbers of activated γδ T cells (IL-17^+^), increased greatly among the IFN-γ^-/-^ mice after immunization (**Figures 6C, D**).

We also compared antigen-specific T cell responses between CD3^+^ T cells isolated from IFN-γ^-/-^ and wt-B6 mice, respectively. The results of crisscross tests, in which the T cells derived from immunized IFN-γ^-/-^ or B6 mice were stimulated either by splenic APCs of irradiated B6 mice or IFN-γ^-/-^ mice (**Figure 6E**) revealed that IFN-γ^-/-^ T cells produced significantly greater amounts of IL-17 when compared to B6 T cells. In addition, IFN-γ^-/-^ splenic APCs had stronger stimulating activity compared to B6 splenic APCs, suggesting that APC function is also augmented in IFN-γ^-/-^ mice.

**Figure 7:**
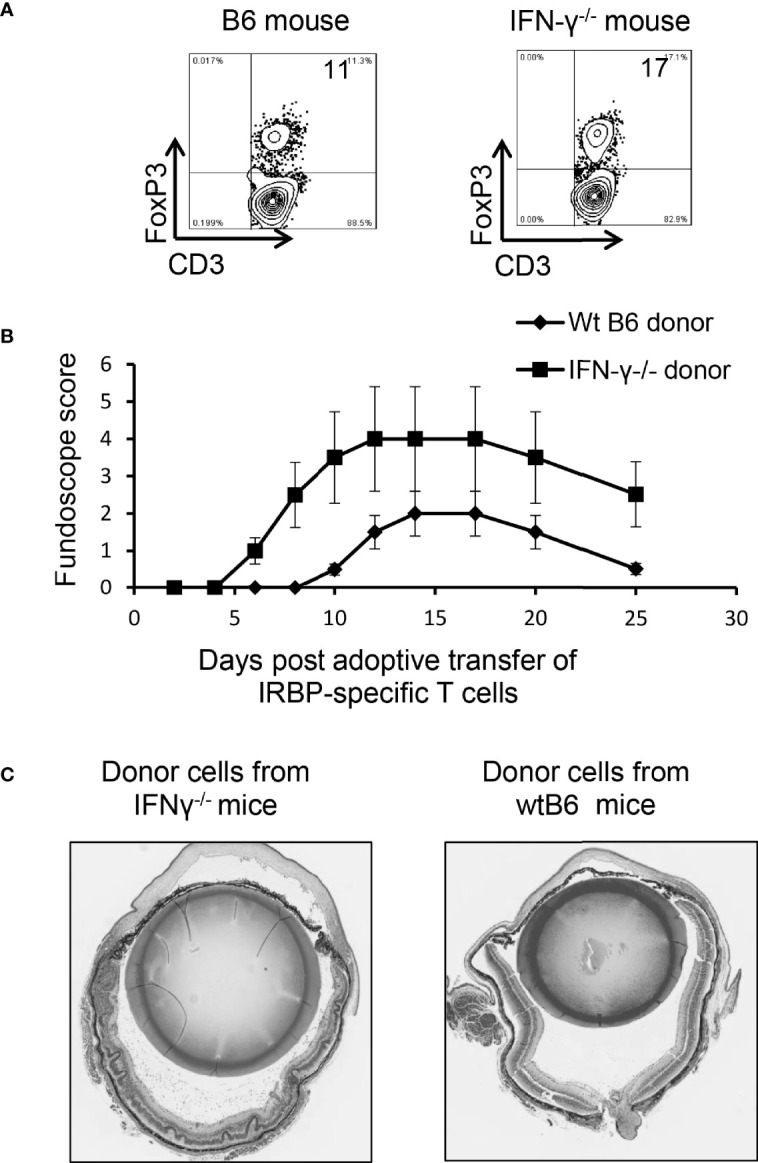
Multiple aberrant immune responses in IFN-γ^-/-^ mice. Foxp3^+^ T cell expansion is increased in IFN-γ^-/-^ mice. In a 24-well plate, the CD3^+^ responder T cells (1.5 x 10^6^/well) derived from B6 mice or IFN-γ^-/-^ mice were cultured in medium containing a very low dose of IL-2 (1ng/ml) for 5 d, which preferentially favors Foxp3^+^ T cell expansion (37). The percentage of Foxp3^+^ T cells among αβTCR^+^ cells was determined by FACS analysis **(A)**. Data are from one single experiment, which is representative of three independent experiments. IRBP-specific T cells isolated from immunized IFN-γ^-/-^ mice have stronger pathogenic activity after adoptive transfer to naïve B6 recipients. IRBP-specific T cells were prepared from the responder T cells of IFN-γ^-/-^ mice and B6 mice. After a 2-d *in vitro* stimulation with the immunizing antigen and splenic APCs. 2 x 10^6^ cells were adoptively transferred to naïve B6 recipient mice *via* i.p. injection **(B)**. Pathologic examination. H&E histologic sections from an eye in each group were obtained on day 30 post-immunization. Severe vitritis and chorioretinal folds occur in IFN-γ^-/-^ mice compared to minimal vitreous inflammation and a normal retina in wt-B6 mice **(C)**.

When Foxp3^+^ T cell responses of wt-B6 and IFN-γ^-/-^ mice were compared, we found that the number of Foxp3^+^ T cells was significantly higher among CD3^+^ T cells in IFN-γ^-/-^ mice (**Figure 7A**). However, the EAU-inducing activity of IRBP-specific T cells isolated from IFN-γ^-/-^ mice and from B6 mice was compared after a 2-d *in vitro* stimulation with the immunizing antigen and splenic APCs by adoptive transfer to naïve B6 mice. IRBP-specific T cells isolated from immunized IFN-γ^-/-^ mice had significantly stronger EAU-inducing activity compared to those from immunized B6 mice (**Figures 7B, C**). These results suggest that augmented Th17 responses in IFN-γ-deficient mice are associated with multiple aberrant immune cell functions or that the increased number of Th17 cells in IFN-γ^-/-^ exceeded the regulatory capacity of Tregs.

## Discussion

IFN-γ production is a hallmark of T helper (Th)1 responses (1, 2) and exerts diverse effects on immune responsiveness. It strengthens innate immunity *via* induction of antimicrobial factors and degradative pathways in other immune cells, such as macrophages; it also augments the antigen-processing and antigen-presenting ability of APCs, stimulates antibody production by B cells, induces the expression of cytokines and chemokines required for the recruitment of myeloid cells to the site of inflammation, and increases the expression of TLRs, NOS, and phagocyte oxidase by macrophages (38). Previous studies demonstrated that the effect of IFN-γ on immune responses could be either pro- (19, 39–42) or anti-inflammatory (23, 24, 43, 44). Elucidation of the bidirectional functions of IFN-γ should increase our understanding of the regulated Th17 responses and their IFN-γ mediated immunomodulation.

Early studies of IFN-γ revealed it to be a key pathogenic molecule in human multiple sclerosis (MS) and in animal models, i.e., experimental autoimmune encephalitis (EAE) (39, 40). Clinical studies showed that MS patients treated with IFN-γ exhibited exacerbated symptoms (42), whereas MS patients treated with antibodies against IFN-γ exhibited reduced clinical symptoms (19). However, a protective effect of IFN-γ has also been demonstrated. Mice deficient in the IFN-γ gene showed an increased incidence of EAE, with earlier disease onset and more severe symptoms (45, 46). Injection of neutralizing antibodies to IFN-γ exacerbated both actively and passively induced EAE (43, 44). Injection of IFN-γ to EAE-prone mice reduced the severity of disease symptoms and mortality (43, 47). A similar protective effect of IFN-γ was also found in other autoimmune diseases, including collagen-induced arthritis, EAU, autoimmune nephritis, and myocarditis (43, 45, 48–52), underscoring the complex role of IFN-γ in disease pathogenesis. Disease stage-specific effects of IFN-γ were also observed. For example, administration of IFN-γ to EAE mice during the inductive period led to disease exacerbation, while a similar treatment during the effector phase was protective (18–22, 53).

Although it has been well established that both Th1 and Th17 autoreactive T cells are pathogenic in various autoimmune diseases (24, 36, 54, 55), including autoimmune uveitis (56), it remained largely undetermined whether therapeutic treatments directed at Th1 responses would also be effective in treating Th17 mediated disease. Our current results demonstrated that IFN-γ is also an effective molecule modulating Th17 responses, although its effect is bidirectional.

The aims of our study were to determine the regulatory mechanisms of Th17 responses in autoimmune diseases. Based on our previous report that the protective effect of adenosine-based treatments is dependent on “timing” ─ ADA was protective when administered during the active phase of EAU but ineffective if administered prior to intraocular inflammation (27). Subsequently, we found that A2AR antagonist SCH58261 (SCH) effectively modulates aberrant Th17 responses in induced EAU. Likewise, timing of the treatment is important (28). We therefore questioned whether such a timing effect applied to other immunoregulatory events. Our results showed that the therapeutic effects differed greatly for Th1 and Th17 responses. Anti-IFN-γ treatment can effectively modulate Th17 responses, albeit in a biphasic fashion. In early phases of EAU, the disease is inhibited by neutralizing IFN-γ activity, whereas during active and ongoing phases of EAU, IFN-γ inhibited disease development by inhibiting Th17 effector T cell responses. Our studies raised an important issue is treating pathogenic T cell responses, particularly the Th17 responses, by showing that an immunological manipulation will be more effective if a “timing” factor has been taken into consideration.

Comparing various immune responses of IFN-γ^-/-^ and B6 mice showed that γδ T cells are overly active in IFN-γ^-/-^ mice, and that IRBP-specific T cells isolated from immunized IFN-γ^-/-^ mice have stronger EAU-inducing activity. Moreover, splenic APCs separated from IFN-γ^-/-^ mice have stronger T cell stimulating activity, indicating that augmented Th17 responses seen in IFN-γ^-/-^ mice are the result of altered immune modulators, leading to delayed onset of disease with later exacerbation. The enhancement or inhibition of EAU correlates with γδ T cell activation. In previous studies we showed that a “timing effect” is a hallmark of adenosine-based immunotherapy. In the current study we showed that the immunomodulatory effect of IFN-γ also involves both enhancement and inhibition of the Th17 response in a “timing effect”. Continued efforts to elucidate the mechanisms underlying the influence of IFN-γ on immune responsiveness eventually should enable us to achieve desired effects, and thus bring us closer to the therapeutic goal of IFN-γ-based treatment of diseases.

## Data Availability Statement

The raw data supporting the conclusions of this article will be made available by the authors, without undue reservation.

## Ethics Statement

The experimental protocols were approved by the Institutional Animal Care and Use Committee of the University of California Los Angeles (Protocol number: ARC#2014-029-03A).

## Author Contributions

DS, HK, and HS designed research. DS and HS performed the experiments and analyzed data. DS and HK wrote the manuscript. All authors contributed to the article and approved the submitted version.

## Funding

This work was supported by NIH grants EY0022403 and EY018827 and grant from for Research to Prevent Blindness, NYC.

## Conflict of Interest

The authors declare that the research was conducted in the absence of any commercial or financial relationships that could be construed as a potential conflict of interest.

## Publisher’s Note

All claims expressed in this article are solely those of the authors and do not necessarily represent those of their affiliated organizations, or those of the publisher, the editors and the reviewers. Any product that may be evaluated in this article, or claim that may be made by its manufacturer, is not guaranteed or endorsed by the publisher.

## References

[1] Mosmann a TR Coffman RL. TH1 and TH2 Cells: Different Patterns of Lymphokine Secretion Lead to Different Functional Properties. Annu Rev Immunol (1989) 7:145–73. doi: 10.1146/annurev.iy.07.040189.001045 2523712

[2] Mosser DM Edwards JP. Exploring the Full Spectrum of Macrophage Activation. Nat Rev Immunol (2008) 8:958–69. doi: 10.1038/nri2448 PMC2724991 19029990

[3] Kalbasi A Tariveranmoshabad M Hakimi K Kremer S Campbell KM Funes JM. Uncoupling Interferon Signaling and Antigen Presentation to Overcome Immunotherapy Resistance Due to JAK1 Loss in Melanoma. Sci Transl Med (2020) 12:eabb0152. doi: 10.1126/scitranslmed.abb0152 33055240 PMC8053376

[4] Shachar I Karin N. The Dual Roles of Inflammatory Cytokines and Chemokines in the Regulation of Autoimmune Diseases and Their Clinical Implications. J Leukoc Biol (2013) 93:51–61. doi: 10.1189/jlb.0612293 22949334

[5] Presti RM Pollock JL Dal Canto AJ O’Guin AK Virgin HW IV. Interferon γ Regulates Acute and Latent Murine Cytomegalovirus Infection and Chronic Disease of the Great Vessels. J Exp Med (1998) 188:577–88. doi: 10.1084/jem.188.3.577 PMC2212470 9687534

[6] Huang S Hendriks W Althage A Hemmi S Bluethmann H Kamijo R. Immune Response in Mice That Lack the Interferon-Gamma Receptor. Science (1993) 259:1742–5. 10.1126/science.8456301 8456301

[7] Harty JT Bevant MJ. Specific Immunity to Listeria Monocytogenes in the Absence of Ifnγ. Immunity (1995) 3:109–17. doi: 10.1016/1074-7613(95)90163-9 7621071

[8] Refaeli Y Van Parijs L Alexander SI Abbas AK. Interferon-G Is Required for Activation-Induced Death of T Lymphocytes. J Exp Med (2002) 196:999–1005. 12370261 10.1084/jem.20020666 PMC2194022

[9] Boehm U Guethlein L Klamp T Ozbek K Schaub A Fütterer A. Two Families of GTPases Dominate the Complex Cellular Response to IFN-γ. J Immunol (1998) 161:6715–23. 9862701

[10] Frucht DM Fukao T Bogdan C Schindler H O’Shea JJ Koyasu S. IFN-γ Production by Antigen-Presenting Cells: Mechanisms Emerge. Trends Immunol (2001) 22:556–60. doi: 10.1016/s1471-4906(01)02005-1 11574279

[11] Fultz MJ Barber SA Dieffenbach CW Vogel SN. Induction of IFN-γ in Macrophages by Lipopolysaccharide. Int Immunol (1993) 5:1383–92. doi: 10.1093/intimm/5.11.1383 8260452

[12] Di Marzio P Puddu P Conti L Belardelli F Gessani S. Interferon Gamma Upregulates Its Own Gene Expression in Mouse Peritoneal Macrophages. J Exp Med (1994) 179:1731–6. doi: 10.1084/jem.179.5.1731 PMC2191486 8163951

[13] Vremec D O’Keeffe M Hochrein H Fuchsberger M Caminschi I Lahoud M. Production of Interferons by Dendritic Cells, Plasmacytoid Cells, Natural Killer Cells, and Interferon-Producing Killer Dendritic Cells. Blood (2006) 109:1165–73. doi: 10.1182/blood-2006-05-015354 17038535

[14] Hochrein H Shortman K Vremec D Scott B Hertzog P O’Keeffe M. Differential Production of IL-12, IFN-α, and IFN-γ by Mouse Dendritic Cell Subsets. J Immunol (2001) 166:5448–55. doi: 10.4049/jimmunol.166.9.5448 11313382

[15] Ohteki T Fukao T Suzue K Maki C Ito M Nakamura M. Interleukin 12–Dependent Interferon γ Production by CD8α+Lymphoid Dendritic Cells. J Exp Med (1999) 189:1981–6. doi: 10.1084/jem.189.12.1981 PMC2192968 10377194

[16] Wohler JE Smith SS Zinn KR Bullard DC Barnum SR. Gd T Cells in EAE: Early Trafficking Events and Cytokine Requirements. Eur J Immunol (2009) 39:1516–26. 10.1002/eji.200839176 PMC2837942 19384874

[17] Ponomarev ED Dittel BN. Gd T Cells Regulate the Extent and Duration of Inflammation in the Central Nervous System by a Fas Ligand-Dependent Mechanism. J Immunol (2005) 174:4678–87. 10.4049/jimmunol.174.8.4678 15814692

[18] Dungan LS McGuinness NC Boon L Lynch MA Mills KHG. Innate IFN-γ Promotes Development of Experimental Autoimmune Encephalomyelitis: A Role for NK Cells and M1 Macrophages. Eur J Immunol (2014) 44:2903–17. doi: 10.1002/eji.201444612 25056715

[19] Arellano G Ottum PA Reyes LI Burgos PI Naves R. Stage-Specific Role of Interferon-Gamma in Experimental Autoimmune Encephalomyelitis and Multiple Sclerosis. Front Immunol (2015) 6:492. doi: 10.3389/fimmu.2015.00492 26483787 PMC4586507

[20] Chu CQ Wittmer S Dalton DK. Failure to Suppress the Expansion of the Activated CD4 T Cell Population in Interferon G-Deficient Mice Leads to Exacerbation of Experimental Autoimmune Encephalomyelitis. J Exp Med (2000) 192:123–8. 10.1084/jem.192.1.123 PMC1887710 10880533

[21] Heremans H Dillen C Groenen M Martens E Billiau A. Chronic Relapsing Experimental Autoimmune Encephalomyelitis (CREAE) in Mice: Enhancement by Monoclonal Antibodies Against Interferon-G. Eur J Immunol (1996) 26:2393–8. 10.1002/eji.1830261019 8898951

[22] Wildbaum G Zohar Y Karin N. Antigen-Specific CD25^–^Foxp3^–^IFN-γ^high^cd4^+^ T Cells Restrain the Development of Experimental Allergic Encephalomyelitis by Suppressing Th17. Am J Pathol (2010) 176:2764–75. doi: 10.2353/ajpath.2010.090855 PMC2877838 20382706

[23] Stromnes IM Cerretti LM Liggitt D Harris RA Goverman JM. Differential Regulation of Central Nervous System Autoimmunity by T(H)1 and T(H)17 Cells. Nat Med (2008) 14:337–42. doi: 10.1038/nm1715 PMC2813727 18278054

[24] Luger D Silver PB Tang J Cua D Chen Z Iwakura Y. Either a Th17 or a Th1 Effector Response Can Drive Autoimmunity: Conditions of Disease Induction Affect Dominant Effector Category. J Exp Med (2008) 205:799–810. 18391061 10.1084/jem.20071258 PMC2292220

[25] Park H Li Z Yang XO Chang SH Nurieva R Wang YH. A Distinct Lineage of CD4 T Cells Regulates Tissue Inflammation by Producing Interleukin 17. Nat Immunol (2005) 6:1133–41. 10.1038/ni1261 PMC1618871 16200068

[26] Harrington LE Hatton RD Mangan PR Turner H Murphy TL Murphy KM. Interleukin 17-Producing CD4+ Effector T Cells Develop via a Lineage Distinct From the T Helper Type 1 and 2 Lineages. Nat Immunol (2005) 6:1123–32. 10.1038/ni1254 16200070

[27] Liang D Zuo A Zhao R Shao H Kaplan HJ Sun D. Regulation of Adenosine Deaminase on Induced Mouse Experimental Autoimmune Uveitis. J Immunol (2016) 196:2646–54. doi: 10.4049/jimmunol.1502294 PMC4779687 26856700

[28] Ko MK Shao H Kaplan HJ Sun D. Timing Effect of Adenosine-Directed Immunomodulation on Mouse Experimental Autoimmune Uveitis. J Immunol (2021) 207:153–61. doi: 10.4049/jimmunol.2100182 PMC8669050 34127521

[29] Cui Y Shao H Lan C Nian H O’Brien RL Born WK. Major Role of Gd T Cells in the Generation of IL-17^+^ Uveitogenic T Cells. J Immunol (2009) 183:560–7. 10.4049/jimmunol.0900241 PMC4077214 19542467

[30] Nian H Shao H O’Brien RL Born WK Kaplan HJ Sun D. Activated Gd Cells Promote the Activation of Uveitogenic T Cells and Exacerbate EAU Development. Invest Ophthalmol Vis Sci (2011) 52:5920–7. 10.1167/iovs.10-6758 PMC3262554 21296823

[31] Liang D Zuo A Shao H Born WK O’Brien RL Kaplan HJ. IL-23 Receptor Expression on γδ T Cells Correlates With Their Enhancing or Suppressive Effects on Autoreactive T Cells in Experimental Autoimmune Uveitis. J Immunol (2013) 191:1118–25. doi: 10.4049/jimmunol.1300626 PMC3720691 23797670

[32] Liang D Zuo A Zhao R Shao H Born WK O’Brien RL. CD73 Expressed on Gd T Cells Shapes Their Regulatory Effect in Experimental Autoimmune Uveitis. PloS One (2016) 11:e0150078. doi: 10.1371/journal.pone.0150078 26919582 PMC4769068

[33] Ko M Shao H Kaplan H Sun D. “Timing Effect” of Adenosine-Directed Immunomodulation on Mouse Experimental Autoimmune Uveitis. J Immunol (2021) 207:153–61. 10.4049/jimmunol.2100182 PMC8669050 34127521

[34] Thurau SR Chan CC Nussenblatt RB Caspi RR. Oral Tolerance in a Murine Model of Relapsing Experimental Autoimmune Uveoretinitis (EAU): Induction of Protective Tolerance in Primed Animals. Clin Exp Immunol (1997) 109:370–6. 10.1046/j.1365-2249.1997.4571356.x PMC1904752 9276535

[35] Nian H Shao H Zhang G Born WK O’Brien R Kaplan HJ. Regulatory Effect of Gd T Cells on IL-17^+^ Uveitogenic T Cells. Invest Ophthalmol Vis Sci (2010) 51:4661–7. 10.1167/iovs.09-5045 PMC2941184 20375337

[36] Peng Y Han G Shao H Wang Y Kaplan HJ Sun D. Characterization of IL-17+ Interphotoreceptor Retinoid-Binding Protein-Specific T Cells in Experimental Autoimmune Uveitis. Invest Ophthalmol Vis Sci (2007) 48:4153–61. doi: 10.1167/iovs.07-0251 PMC2567912 17724201

[37] Liang D Woo J-I Shao H Born WK O’Brien RL Kaplan HJ. Ability of γδ T Cells to Modulate the Foxp3 T Cell Response Is Dependent on Adenosine. PloS One (2018) 13:e0197189. doi: 10.1371/journal.pone.0197189 29771938 PMC5957379

[38] Hu X Chakravarty SD Ivashkiv LB. Regulation of Interferon and Toll-Like Receptor Signaling During Macrophage Activation by Opposing Feedforward and Feedback Inhibition Mechanisms. Immunol Rev (2008) 226:41–56. doi: 10.1111/j.1600-065X.2008.00707.x 19161415 PMC2630590

[39] Nylander A Hafler DA. Multiple Sclerosis. J Clin Invest (2012) 122:1180–8. doi: 10.1172/JCI58649 PMC3314452 22466660

[40] Kuchroo VK Anderson AC Waldner H Munder M Bettelli E Nicholson LB. T Cell Response in Experimental Autoimmune Encephalomyelitis (EAE): Role of Self and Cross-Reactive Antigens in Shaping, Tuning, and Regulating the Autopathogenic T Cell Repertoire. Ann Rev.Immunol (2002) 20:101–23. doi: 10.1146/annurev.immunol.20.081701.141316 11861599

[41] Panitch H Haley A Hirsch R Johnson K. Exacerbations of Multiple Sclerosis in Patients Treated With Gamma Interferon. Lancet (1987) (8538):893–5. doi: 10.1016/S0140-6736(87)92863-7 2882294

[42] Panitch HS Heracl RI Schindler J Johnson K. Treatment of Multiple Sclerosis With Gamma Interferon: Exacerbations Associated With Activation of the Immune System. Neurology (1987) 37:1097–112. 10.1212/wnl.37.7.1097 3110648

[43] Billiau A Heremans H Vandekerckhove F Dijkmans R Sobis H Meulepas E. Enhancement of Experimental Allergic Encephalomyelitis in Mice by Antibodies Against IFN-G. J Immunol (1988) 140:1506–10. 3126227

[44] Duong TT Finkelman FD Singh B Strejan GH. Effect of Anti-Interferon-Gamma Monoclonal Antibody Treatment on the Development of Experimental Allergic Encephalomyelitis in Resistant Mouse Strains. J Neuroimmunol (1994) 53:101–7. 10.1016/0165-5728(94)90069-8 8051292

[45] Ferber IA Brocke S Taylor-Edwards C Ridgway W Dinisco C Steinman L. Mice With a Disrupted IFN-G Gene Are Susceptible to the Induction of Experimental Autoimmune Encephalomyelitis (EAE). J Immunol (1996) 156:5–7. 8598493

[46] Krakowski M Owens T. Interferon-G Confers Resistance to Experimental Allergic Encephalomyelitis. Eur J Immunol (1996) 26:1641–6. 10.1002/eji.1830260735 8766573

[47] Voorthuis JA Uitdehaag BM De Groot CJ Goede PH van der Meide PH Dijkstra CD. Suppression of Experimental Allergic Encephalomyelitis by Intraventricular Administration of Interferon-Gamma in Lewis Rats. Clin Exp Immunol (1990) 81:183–8. doi: 10.1111/j.1365-2249.1990.tb03315.x PMC1535058 2117508

[48] Willenborg DO Fordham S Bernard CCA Cowden WB Ramshaw IA. IFN-G Plays a Critical Down-Regulatory Role in the Induction and Effector Phase of Myelin Oligodendrocyte Glycoprotein-Induced Autoimmune Encephalomyelitis. J Immunol (1996) 157:3223–7. 8871615

[49] Jones LS Rizzo LV Agarwal RK Tarrant TK Chan CC Wiggert B. IFN-Gamma-Deficient Mice Develop Experimental Autoimmune Uveitis in the Context of a Deviant Effector Response. J Immunol (1997) 158:5997–6005. 9190954

[50] Caspi RR Chan CC Grubbs BG Silver PB Wiggert B Parsa CF. Endogenous Systemic IFN-G has a Protective Role Against Ocular Autoimmunity in Mice. J Immunol (1994) 152:890–9. 8283058

[51] Ring GH Dai Z Saleem S Baddoura FK Lakkis FG. Increased Susceptibility to Immunologically Mediated Glomerulonephritis in IFN-γ-Deficient Mice. J Immunol (1999) 163:2243–8. 10438967

[52] Eriksson U Kurrer MO Sebald W Brombacher F Kopf M. Dual Role of the IL-12/IFN-γ Axis in the Development of Autoimmune Myocarditis: Induction by IL-12 and Protection by IFN-γ. J Immunol (2001) 167:5464–9. doi: 10.4049/jimmunol.167.9.5464 11673566

[53] Naves R Singh SP Cashman KS Rowse AL Axtell RC Steinman L. The Interdependent, Overlapping, and Differential Roles of Type I and II IFNs in the Pathogenesis of Experimental Autoimmune Encephalomyelitis. J Immunol (2013) 191:2967–77. doi: 10.4049/jimmunol.1300419 PMC3779698 23960239

[54] Korn T Bettelli E Oukka M Kuchroo VK. IL-17 and Th17 Cells. Annu Rev Immunol (2009) 27:485–517. 19132915 10.1146/annurev.immunol.021908.132710

[55] Kroenke MA Carlson TJ Andjelkovic AV Segal BM. IL-12- and IL-23-Modulated T Cells Induce Distinct Types of EAE Based on Histology, CNS Chemokine Profile, and Response to Cytokine Inhibition. J Exp Med (2008) 205:1535–41. 10.1084/jem.20080159 PMC2442630 18573909

[56] Amadi-Obi A Yu C-R Liu X Mahdi RM Clarke GL Nussenblatt RB. TH17 Cells Contribute to Uveitis and Scleritis and Are Expanded by IL-2 and Inhibited by IL-27/Stat1. Nat Med (2007) 13:711–8. doi: 10.1038/nm1585 17496900

